# Supplementary figures and images for: Arabidopsis CNL receptor SUT1 confers immunity in hydathodes against the vascular pathogen Xanthomonas campestris pv. campestris
Source: PLoS Pathog. 2025 Jun 30;21(6):e1013256. doi: 10.1371/journal.ppat.1013256 (PMC12233906; doi:10.1371/journal.ppat.1013256)

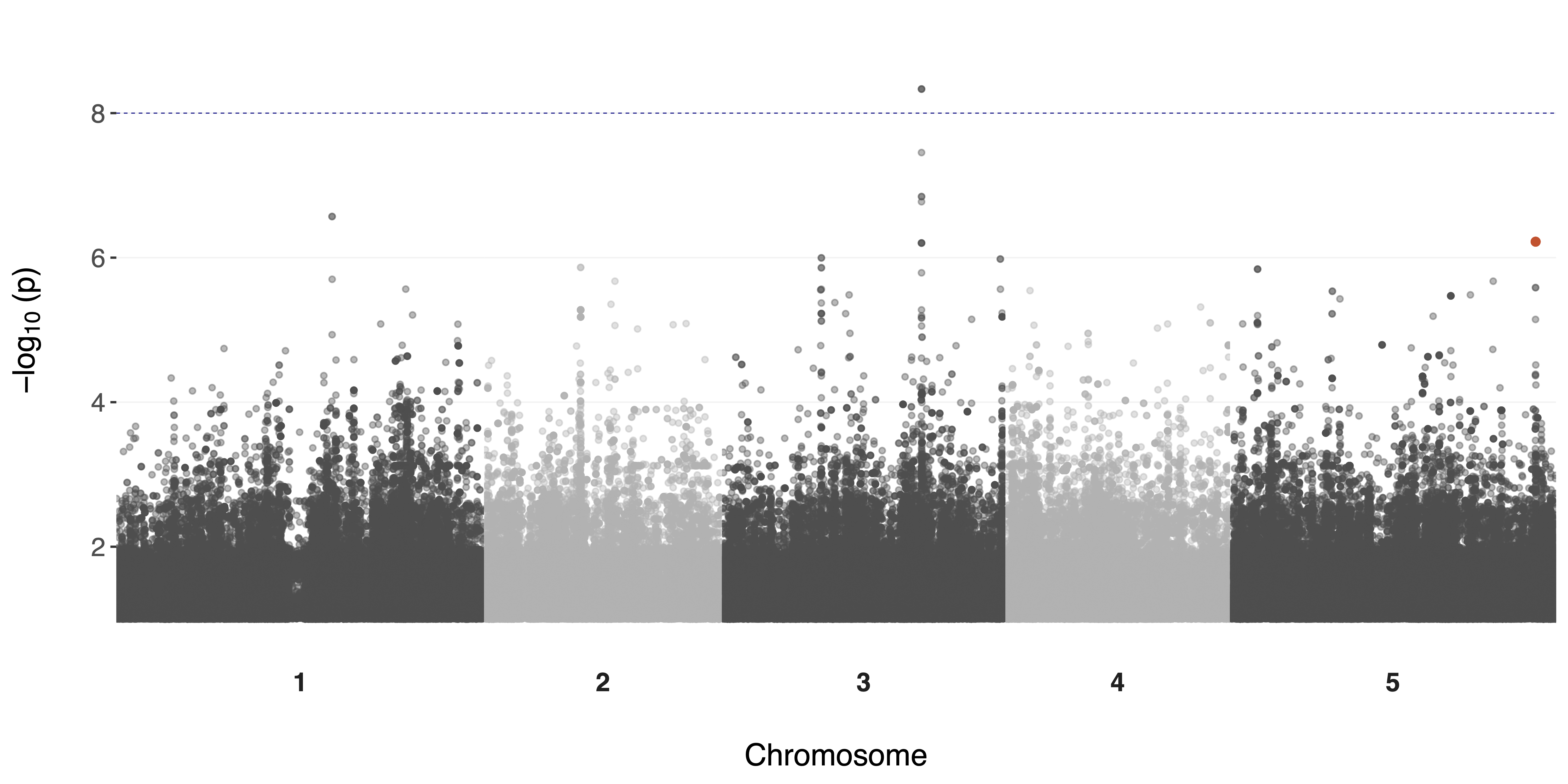

Supplement: S1 Fig — Highlighted SNPs in orange are located within the coding sequence of SUT1. False Discovery Rate (FDR) threshold set at -log10 (p) = 8 (Bonferroni). Significantly correlated SNP on Chr 3 is located in the LAS1 gene (AT3G45130). (TIF) [file ppat.1013256.s001.tif]

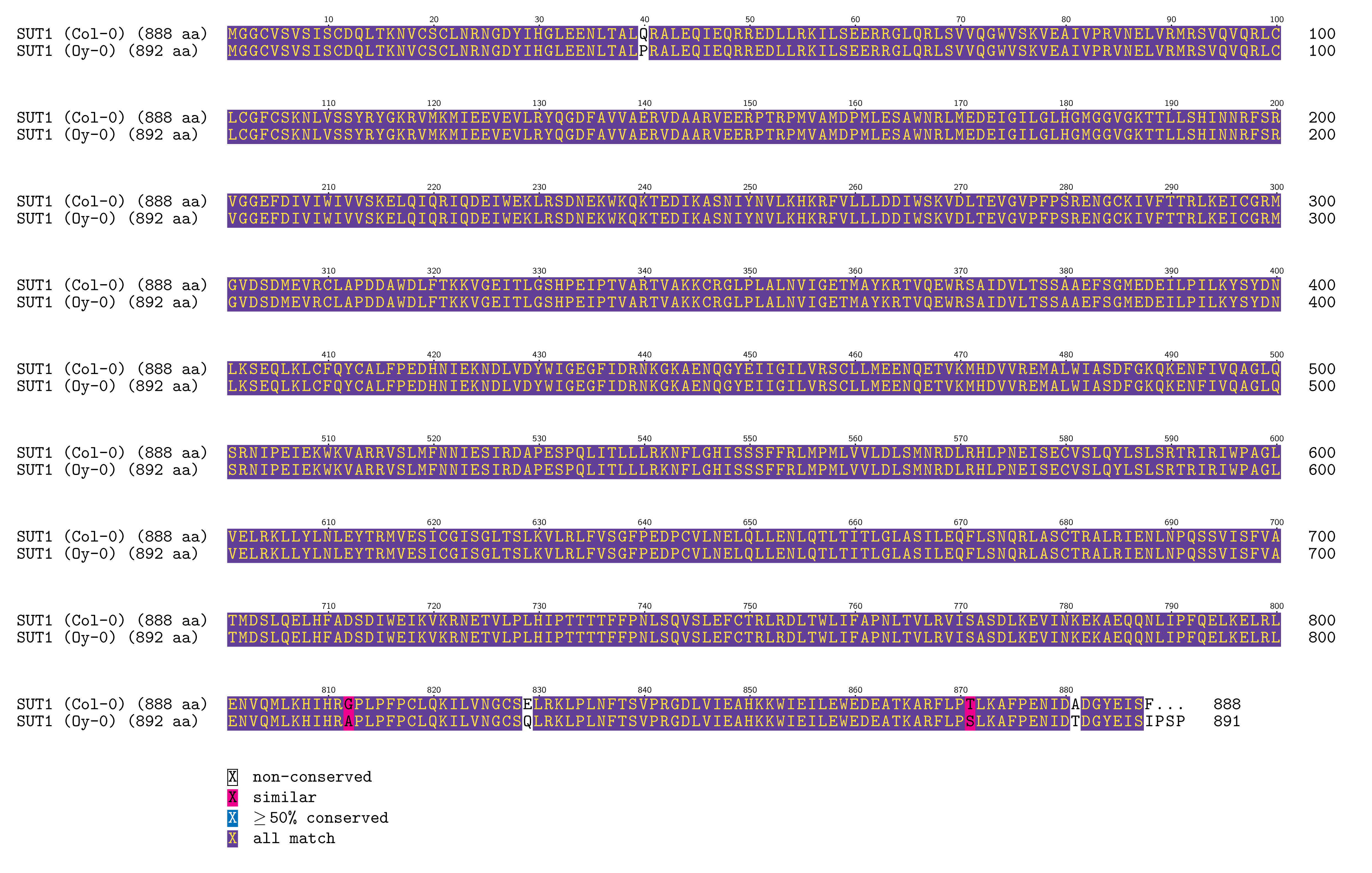

Supplement: S2 Fig — Coding sequences of SUT1Col-0 and SUT1Oy-0 were obtained from [31] and translated in silico into amino acid sequences. Alignment depicts six predicted amino acid substitutions in SUT1Oy-0 compared to SUT1Col-0, of which one is predicted to result in a 3-residue peptide extension at the C-terminus in SUT1Oy-0. (TIF) [file ppat.1013256.s002.tif]

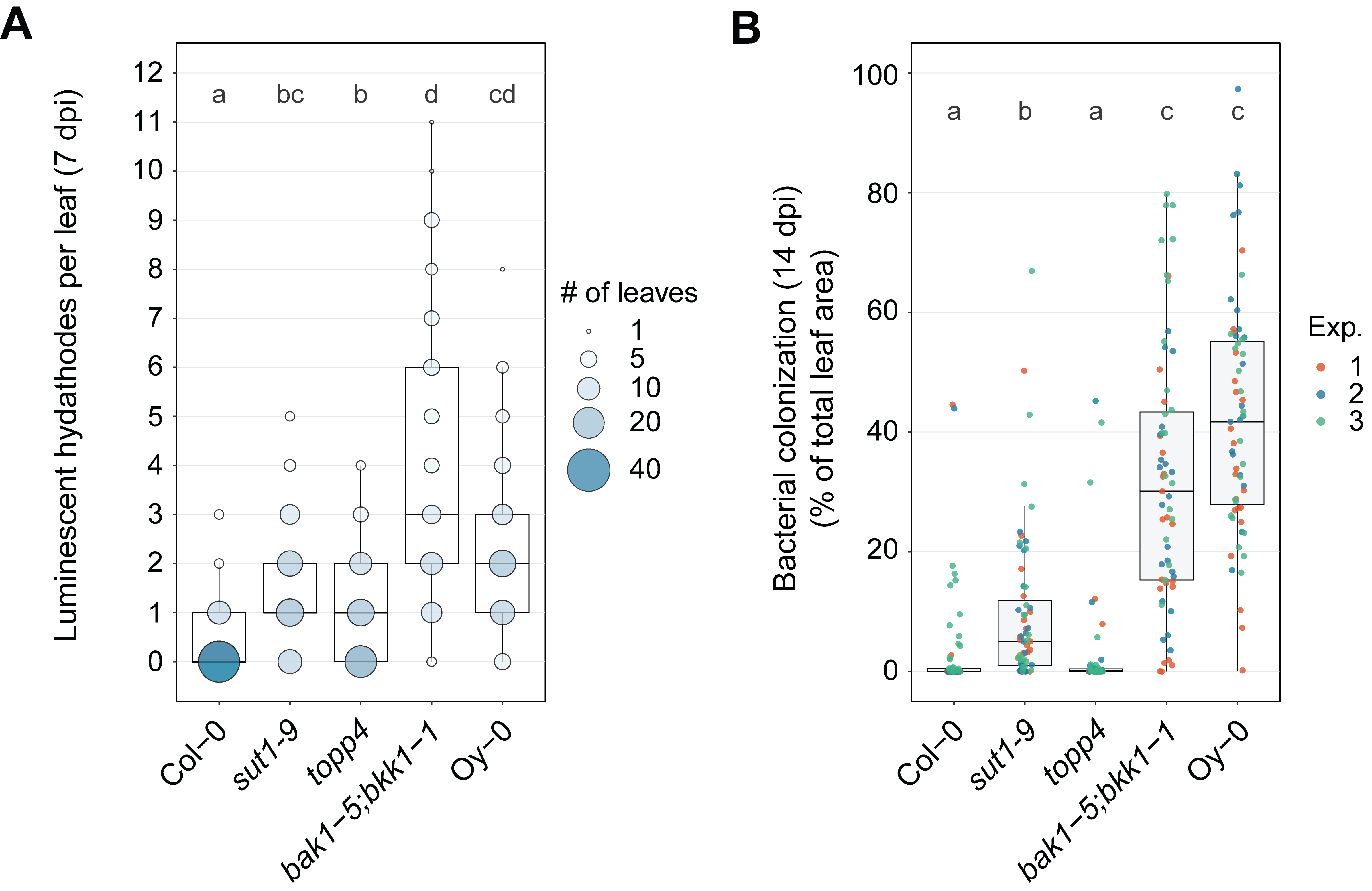

Supplement: S3 Fig — A) Number of infected hydathodes per leaf at 7 dpi following spray inoculation with Xcc8004 ΔxopAC Tn7:lux. Circles depict number of leaves for each score (n = 63 leaves per plant line). The topp4 knockout shows a slight increase in hydathode infections compared to the Col-0 control. Significance letters from non-parametric Kruskal-Wallis test with Dunn’s Post-Hoc test, p-value threshold = 0.05. B) Bacterial colonization defined as percentage of total leaf area colonized per leaf following spray inoculation with Xcc8004 ΔxopAC Tn7:lux (14 dpi, n = 21 leaves per experiment). Bacterial colonization was calculated using ScAnalyzer [58]. The topp4 knockout does not display increased bacterial spread compared to the wildtype control (Col-0). Significance letters from non-parametric Kruskal-Wallis test with Dunn’s Post-Hoc test, p-value threshold = 0.05. (TIF) [file ppat.1013256.s003.tif]

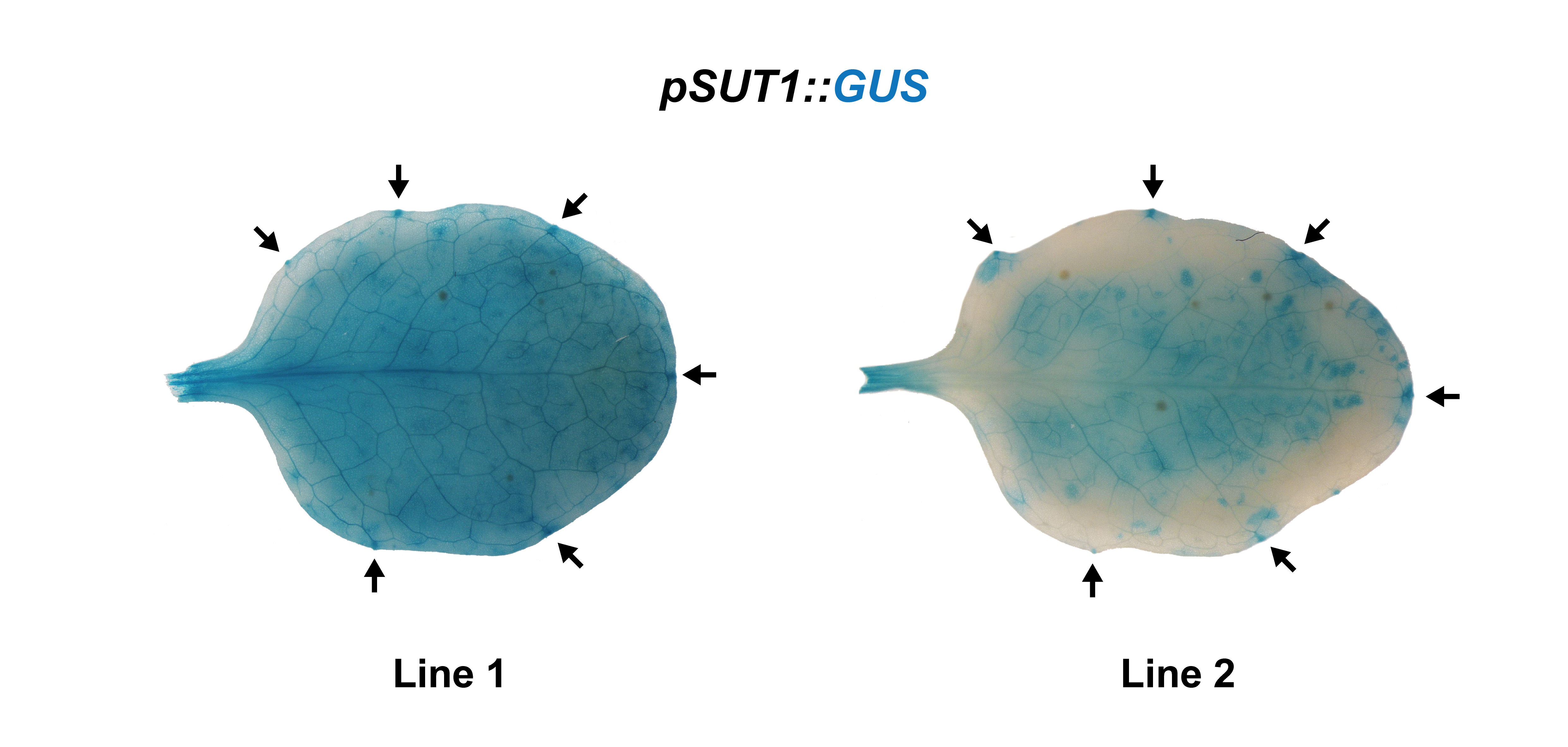

Supplement: S4 Fig — Two independent homozygous T3 lines of 4-week-old Arabidopsis pSUT1::GUS show GUS enzymatic activity in hydathodes (black arrows) and mesophyll tissue throughout the leaf. Leaves were incubated in GUS staining solution for two hours. (TIF) [file ppat.1013256.s004.tif]

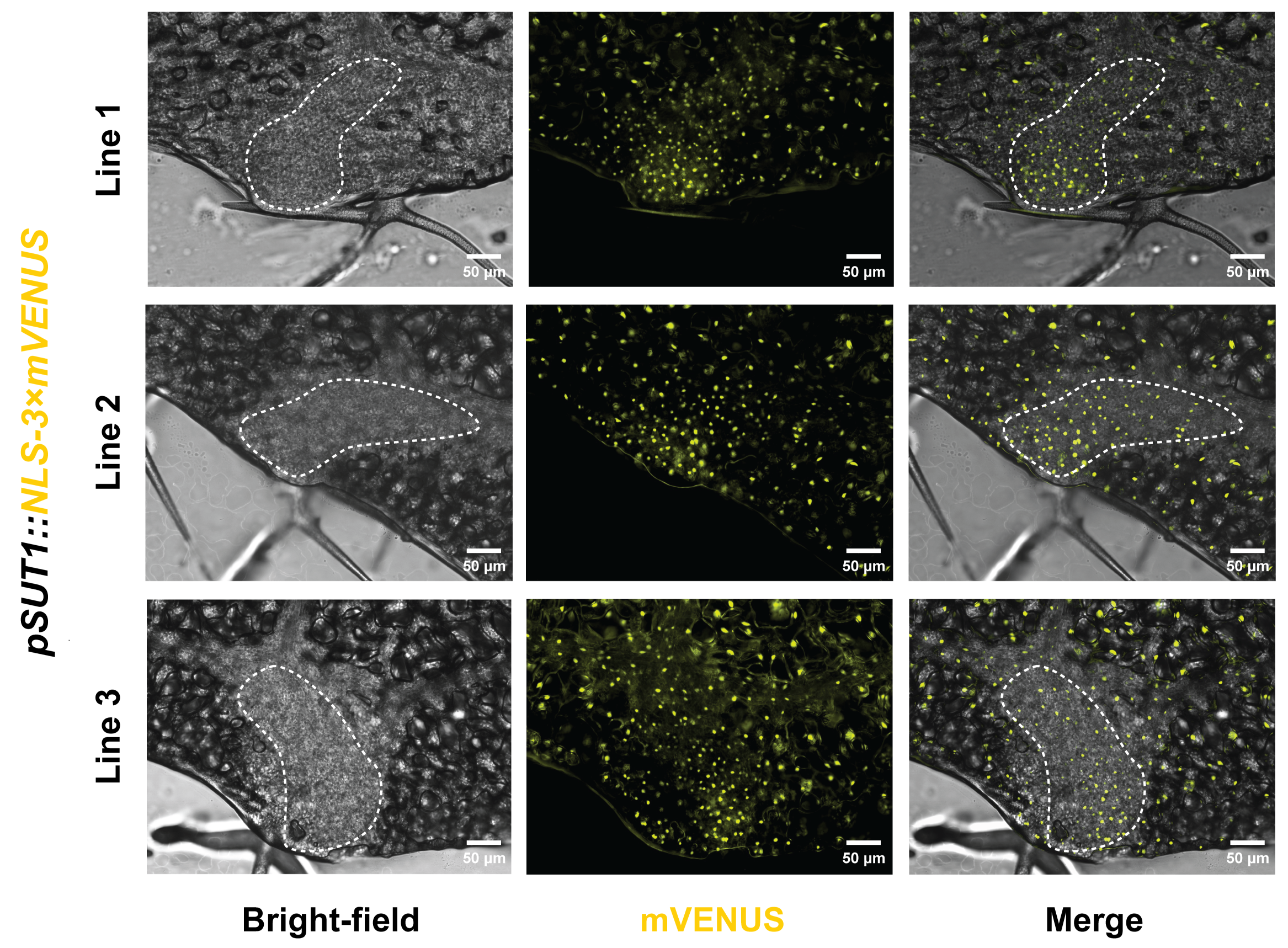

Supplement: S5 Fig — Three independent T2 lines of 4-week-old Arabidopsis pSUT1::NLS-3 × mVENUS show nuclear accumulation of mVENUS in the mesophyll and epithem. Epithem cells can be distinguished from mesophyll cells in the bright-field image as a cell dense area present on the leaf margin connected to the vasculature (white dashed line). (TIF) [file ppat.1013256.s005.tif]

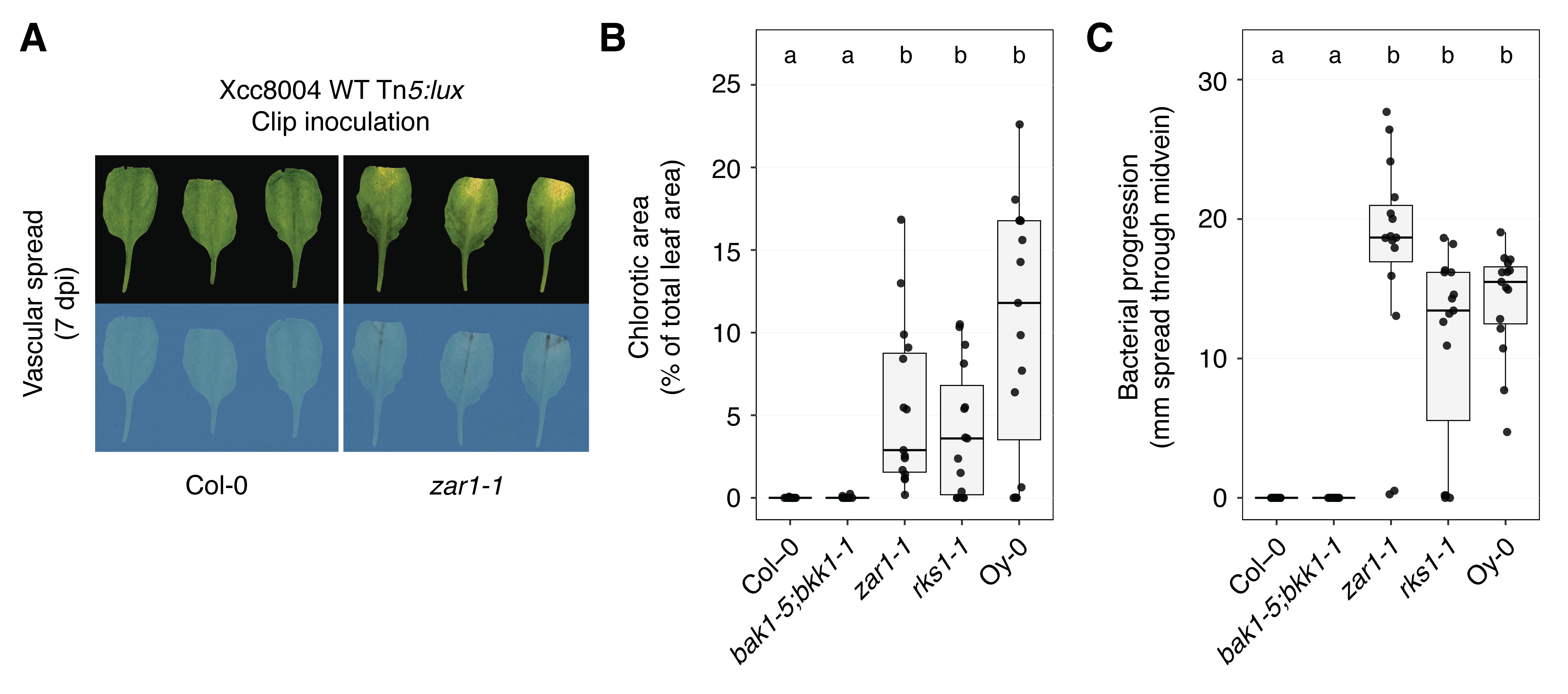

Supplement: S6 Fig — A) Disease symptoms and bacterial luminescence in resistant Col-0 and susceptible zar1–1 knockout lines, 7 dpi following clip inoculation with Xcc8004 WT Tn5:lux. B,C) Chlorotic leaf area (B) and bacterial spread along the midvein (C) 7 dpi following clip inoculation with Xcc8004 WT Tn5:lux on Arabidopsis Col-0, bak1–5;bkk1–1, zar1–1, rks1–1 and Oy-0 (n = 12 leaves). ZAR1-mediated resistance against Xcc is effective in the vasculature of Col-0 and bak1–5;bkk1–1 when the hydathodes are bypassed and the bacteria are introduced using leaf clipping. Significance letters from a two-way ANOVA with Tukey Post-Hoc test, p-value threshold = 0.05. (TIF) [file ppat.1013256.s006.tif]

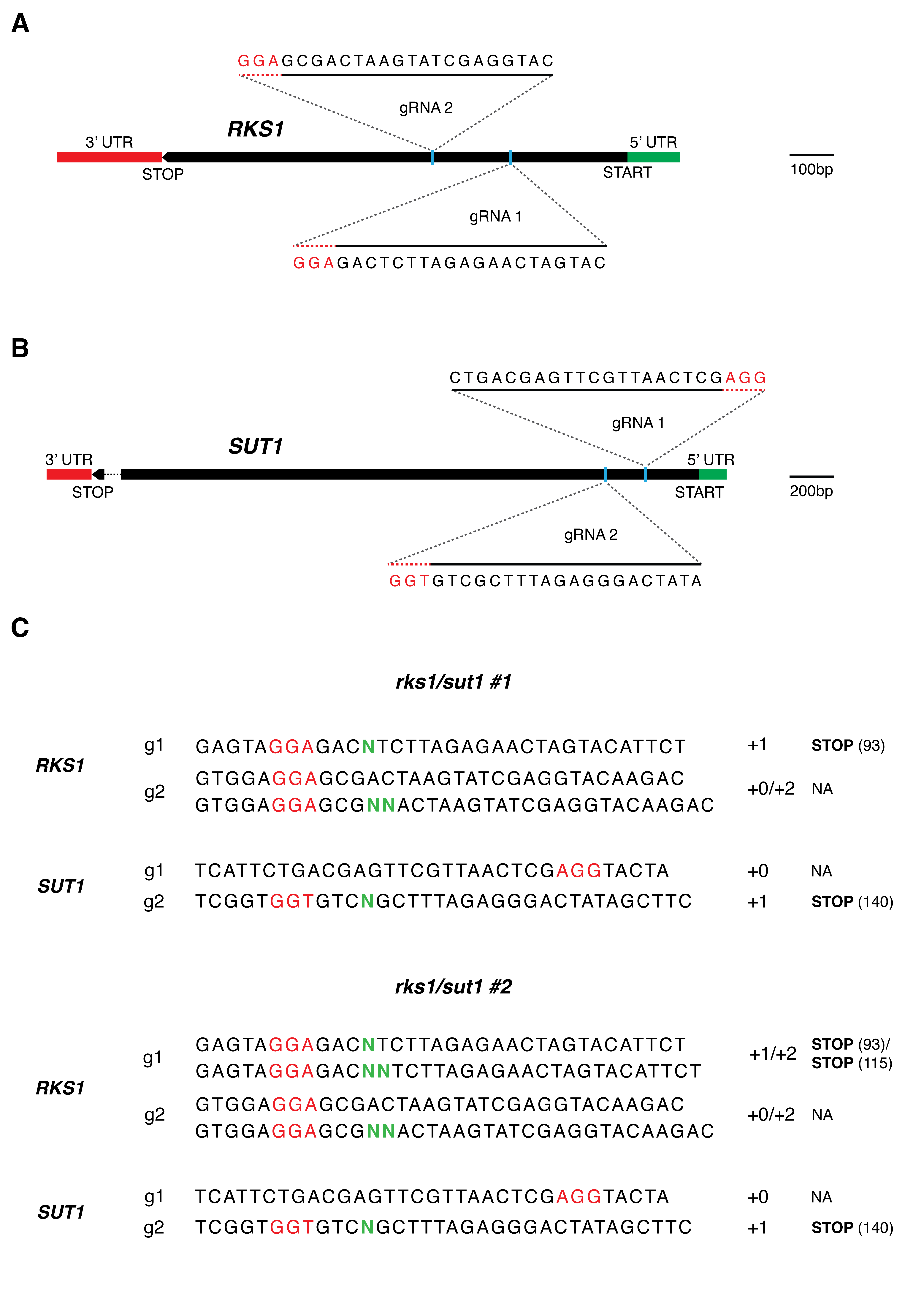

Supplement: S7 Fig — A,B) Schematic overview of two guide RNAs targeting the first exons of each target gene, RKS1 (A) and SUT1 (B), in Col-0. C) Schematic overview of resulting homozygous (when single sequence is shown) or biallelic (when two sequences are shown) indels (green N’s) introduced at each gRNA target sode (g1 and g2) in two individual T1 mutant lines (#1 and #2). Both lines, rks1/sut1 #1 and #2, show confirmed early STOP codons in both alleles of both target genes, as indicated on the right. (TIF) [file ppat.1013256.s007.tif]
